# Two weeks of western diet disrupts liver molecular markers of cholesterol metabolism in rats

**DOI:** 10.1186/s12944-020-01351-2

**Published:** 2020-08-21

**Authors:** Roxane St-Amand, Émilienne T. Ngo Sock, Samantha Quinn, Jean-Marc Lavoie, David H. St-Pierre

**Affiliations:** 1 École de Kinésiologie et des Sciences de l’Activité Physique, Faculté de Médecine, Université de Montréal, Montréal, Canada; 2 Department of Exercise Sciences, Université du Québec à Montréal, 141, Avenue Président-Kennedy, C.P. 8888, succursale Centre-Ville, Montréal, Québec H3C 3P8 Canada

**Keywords:** Western diet, Plasma cholesterol, Lipogenesis, Hepatic steatosis, Liver cholesterol metabolism, Low-density lipoprotein-receptor, Proprotein convertase subtilisin/kexin 9

## Abstract

**Background:**

The present study was designed to test the hypothesis that in the liver, excessive fat accumulation impairs cholesterol metabolism mainly by altering the low-density lipoprotein-receptor (LDL-R) pathway.

**Method:**

Young male Wistar rats were fed standard (SD), high fat (HFD; 60% kcal) or Western (WD; 40% fat + 35% sucrose (17.5% fructose)) diets for 2 or 6 weeks.

**Results:**

Weight gain (~ 40 g) was observed only following 6 weeks of the obesogenic diets (*P* < 0.01). Compared to the 2-week treatment, obesogenic diets tripled fat pad weight (~ 20 vs 7 g) after 6 weeks. Hepatic triglyceride (TG) levels were greater in response to both the WD and HFD compared to the SD (*P* < 0.01) at 2 and 6 weeks and their concentrations were greater (*P* < 0.05) in WD than HFD at 2 weeks. Plasma total cholesterol levels were higher (*P* < 0.05) in animals submitted to WD. After 2 and 6 weeks, liver expression of LDL-R, proprotein convertase subtilisin/kexin 9 (PCSKk9) and sterol regulatory element binding protein 2 (SREBP2), involved in LDL-cholesterol uptake, was lower in animals submitted to WD than in others treated with HFD or SD (*P* < 0.01). Similarly, low-density lipoprotein-receptor-related protein 1 (LRP1) and acyl-CoA cholesterol acyltransferase-2 (ACAT-2) mRNA levels were lower (*P* < 0.01) among WD compared to SD-fed rats. Expression of the gene coding the main regulator of endogenous cholesterol synthesis, 3-hydroxy-3-methyl-glutaryl-CoA reductase (HMGCoAR) was reduced in response to WD compared to SD and HFD at 2 (*P* < 0.001) and 6 (*P* < 0.05) weeks. Being enriched in fructose, the WD strongly promoted the expression of carbohydrate-response element binding protein (ChREBP) and acetyl-CoA carboxylase (ACC), two key regulators of de novo lipogenesis.

**Conclusion:**

These results show that the WD promptly increased TG levels in the liver by potentiating fat storage. This impaired the pathway of hepatic cholesterol uptake via the LDL-R axis, promoting a rapid increase in plasma total cholesterol levels. These results indicate that liver fat content is a factor involved in the regulation of plasma cholesterol.

## Introduction

Non-alcoholic fatty liver disease (NAFLD), also referred to as hepatic steatosis is characterized by excessive fat accumulation (5 to 10% of liver weight) in absence of significant alcohol consumption [1]. This state is associated with several clinical and pathological manifestations. NAFLD is considered a risk factor for diabetes and cardiovascular diseases (CVD) independently of other usual risk factors [2, 3]. Hepatic steatosis by itself is also associated with a proatherogenic lipid profile and production of proinflammatory markers [4, 5]. Some authors [6] have even claimed that with regards to body fat accumulation, hepatocytes are likely the first cell types to display metabolic impairments.

The association between liver fat accumulation, CVD, and a proatherogenic lipid profile is well substantiated in the literature. However, the critical impact of hepatic cholesterol metabolism linking these health conditions remains largely overlooked. The liver is the master regulator of several biological pathways involved in the control of cholesterol metabolism. These include cholesterol synthesis, uptake from chylomicron remnants, re-uptake from LDL and high-density lipoproteins (HDL) as well as the release of cholesterol under very-low density lipoproteins (VLDL), and biliary acids production. All of these pathways have a major influence on the regulation of plasma cholesterol levels and the subsequent proatherogenic profile associated with fat accumulation in the liver.

The association between hepatic steatosis and cholesterol metabolism is most often viewed as how cholesterol metabolism dysregulation contributes to NAFLD in animal models [7]. However, it was also proposed that NAFLD impairs cholesterol metabolism in humans [8]. Long-term studies do not provide any clear indication that fat accumulation in the liver is at the origin of alterations in cholesterol metabolism. On the other hand, several molecular markers of cholesterol metabolism in the liver were disturbed by a short-term exposure (2 weeks) to HFD in young rats [9]. These results support the concept that excessive accumulation of fat in the liver may contribute to increased circulating cholesterol levels [9]. A short-term 2-week HFD was used in that study because it has been shown to result in a rapid fat accumulation in the liver. This excessive accumulation of fat in the liver was previously shown to precede both the occurrence of significant changes in weight gain and the development of systemic metabolic complications classically observed in response to longer exposure to obesogenic diets [10, 11].

The notion that disturbances in lipid metabolism in the liver play a crucial role in the etiology of plasma hypercholesterolemia is supported by the knowledge that TG and cholesterol metabolic pathways are closely interrelated in this organ. Cholesterol and fatty acid synthesis both use acetyl-CoA as biochemical starting material, and the reducing equivalent NADPH as a source of energy for their synthesis. Therefore, there is potential competition between fatty acid synthesis stored as TG and cholesterol synthesis in the liver. This raises the possibility that de novo lipogenesis resulting in liver fat accumulation may impair hepatic cholesterol metabolism. This hypothesis was recently supported by Ichigo and colleagues [12] who reported that in comparison to a high glucose (65%) diet, exposure to a high fructose (65%) diet for 12 days increased ACAT-2 (enzyme acyl-CoA acyl transferase 2: involved in cholesterol esterification) while decreasing ATP-binding cassette protein G5 (ABCG5) and ABCG8 (dimeric proteins involved in the exportation of cholesterol from the liver to bile ducts) gene expression in the liver. The present study tested the hypothesis that a short-term exposure (2 weeks) to two obesogenic diets; a HFD known to rapidly increase fat accumulation in the liver and a WD (40% fat and 35% sucrose (17.5% fructose)) known to stimulate de novo lipogenesis disrupts molecular pathways of cholesterol metabolism in the liver. Among the different pathways of cholesterol metabolism, the important receptor LDL-R involved in cholesterol uptake of LDL particles in plasma was investigated along with its posttranscriptional regulator PCSK9 and their transcription factor SREBP2.

## Material and methods

### Animals care and treatments

Four-week old male Wistar rats (Charles River, St-Constant, Québec, Canada) weighing 190 to 200 g, were housed individually and had ad libitum access to food and tap water after they were received at the animal facility. Their environment was controlled in terms of light (12 h light-dark cycling starting at 06:00 AM), humidity, and room temperature (20–23 °C). After a 3-day acclimatisation period, rats were randomly submitted either to a SD chow diet, a 60% HFD, or a WD (40% fat-35% sucrose) diet for 2 or 6 weeks for a total of 6 experimental groups (*n* = 10/group). Food intake and body weight were measured every 2 and 3 days, respectively. The protocol was approved by the *Comité Institutionnel de Protection des Animaux* (CIPA; 1218–956-1219) of UQAM and developed in agreement with the Canadian Council on Animal Care’s rules (CCAC-CCPA).

At the end of their respective nutritional treatments, rats were euthanized between 09:00 and 11:00 AM after a 3-h fast. Immediately after complete anaesthesia by inhalation of isoflurane (3% induction and 1.5% maintenance) blood samples were drawn from the heart and collected in tubes containing 0.5 ml EDTA. The blood was centrifuged (1500 RPM for 10 min), and the plasma was collected to measure TG, cholesterol, and PCSK9. The abdominal cavity was opened, and the median lobe of the liver was freeze-clamped, removed and used for TG, cholesterol and gene expression of several key markers of cholesterol metabolism. The mesenteric, retroperitoneal, epididymal, and subcutaneous (sc) fat deposits were, thereafter, rapidly excised and weighed. The mesenteric fat pad consisted of adipose tissue surrounding the gastrointestinal tract from the gastroesophageal sphincter to the end of the rectum with special care taken in distinguishing and removing pancreatic cells. The epididymal fat pad included adipose tissue surrounding the ureters and bladder. The retroperitoneal fat pad represented the distinct deposit behind each kidney, along the lumbar muscles. For sc fat deposit measurement, a rectangular piece of skin was taken on the right side of each animal from the median line of the abdomen to the spine and the right hip to the first rib [13]. All tissue and plasma samples were stored at − 78 °C until analyses.

### Diets

Diets were selected to compare short- and mid-term effects of a standard rodent diet (SD) with two types of obesogenic diets enriched in: lipids (HFD) or fat and sucrose (WD). Fat and macronutrient contents were not matched for the selected diets. The energy content (% kcal) of the SD [14] was 13% lipids, 65.6% carbohydrates and 21.4% proteins (Charles River Rodent Diet 5075, Cargill Animal Nutrition, MN, USA). It had a gross energy value of 3970 kcal/kg and a physiological fuel value of 2885 kcal/kg. Its macronutrient composition (g/100 g) was 55.2% carbohydrates (38% glucose derived from corn starch:100% glucose); 13% neutral detergent fiber and 4.1% acid detergent fiber derived from wheat middlings and wheat); 4.5% lipids (poultry fat) and 18% proteins (dehulled soybean meal, fish meal and whey). The HFD and the WD were obtained from Research Diets (NJ, USA). The HFD consisted of 60% fat, 20% CHO and 20% protein (kcal) while the WD consisted of 40% lipid, 43% CHO (35% sucrose), and 17% protein (kcal). The caloric equivalent of 2.89, 5.21 and 4.67 kcal/g for the SD, HFD, and WD, respectively was used to report food intake in kcal. Details of the HFD and WD are presented in Table 1.

**Table 1:** Description of the diets

Ingredients (g)	High Fat diet D12492	Western diet D12079B
Casein, Lactic, 30 Mesh	200	195
Methionine, DL	0	3
Cystine, L	3	0
Sucrose, Fine Granulated	72.8	350
Startch, Corn	0	50
Lodex 10	125	100
Solka Floc, FCC200 (Fiber)	50	50
Butter, Anhydrous	0	200
Corn oil	0	10
Lard	245	0
Soybean Oil, USP	25	0
S10026B (Mineral)	50	0
S10001A (Mineral)	0	17.5
Calcium Phosphate, Dibasic (Mineral)	0	17.5
Calcium Carbonate, Light, USP (Mineral)	0	4
Choline Bitartrate (Vitamin)	2	2
V10001C (Vitamin)	1	1
Ethoxyquin (Anti-oxydant)	0	0.04
Cholesterol, NF	0	1.5
Dye, Blue FD&C #1	0.05	0

### Biochemical analyses

Hepatic TG and cholesterol levels were determined using approximately 100 mg of tissue. Overnight 2:1 chloroform/methanol solution was used to extract lipids. Following centrifugation, nitrogen flux was used to dried up the lower phase and resuspended with Floch and Thesit solution (20%). Quantification of total cholesterol and TG from the liver and plasma was performed using Cholesterol E kits (Wako Diagnostics, Mountain View, CA, USA) and a TG assay (Randox Laboratories Ltd., Crumlin, UK). Plasma PCSK9 concentrations were measured using Mouse/Rat PCSK9 ELISA kits from CircuLex (Cat# CY-8078).

### Real-time polymerase chain reaction

Quick-frozen tissue samples of liver were powdered with a cold pestle and mortar, and ~ 100 mg was used for the isolation of RNA. Total RNA was extracted using RNeasy Micro (Qiagen). RNA integrity was validated using a Bioanalyzer 2100 (Agilent). Total RNA was treated with DNase and reverse transcribed using the Maxima First Strand cDNA synthesis kit with ds DNase (Thermo Fisher Scientific). A BioDrop spectrophotometer was used to determine RNA concentrations, and the ratio of absorbance at 260 and 280 nm was used to assess purity. RNA integrity was evaluated by visualization of intact 18S and 28S RNA bands following agarose gel electrophoresis. SuperScript VILO Master Mix (Invitrogen) was used to synthesize cDNA with 1 μg of RNA per 20 μL reaction.

Real-time qPCR reactions were performed in triplicate using 384-well plates in the QuantStudio6 system (Life Technologies) with SYBR Select Master Mix kit (Applied Biosystems). Reaction conditions consisted of 2 μL of a 1:10 cDNA dilution and 0.3 μM primers in a final volume of 10 μL. The following cycling protocol was used: 2 min at 50 °C, 2 min at 95 °C, followed by 40 cycles of 15 s at 95 °C, 15 s at 58 °C, and 1 min at 72 °C. The reaction’s specificity was verified by melting curve analysis. Negative controls (RT) and no template controls (NTC) were included in the PCR runs. Reference genes were identified by testing 10 different genes (picked from literature) and running TaqMan Array Rat Endogenous Control Plates (Applied Biosystems) on all samples. The 3 reference (housekeeping) genes with the best gene expression stability measure (Expression Suite, Applied Biosystems) were selected for normalization: Hprt, Actb, and Gapdh. Primers used (Table 2) were designed using Primer-BLAST (NCBI). Whenever possible, intron-spanning primers were selected to avoid amplification of genomic DNA. A standard curve obtained by scalar dilution of a cDNA pool was generated to verify PCR efficiency. The triplicate average values of cycle threshold (Ct) were used for quantification, and target genes were determined by 2ΔΔCt calculation method. Results are presented as Arbitrary Units (AU; also reported as Fold Change or Relative Expression in the literature).

**Table 2:** Oligonucleotide primers used for quantitative real-time polymerase chain reaction

*Gene name NCBI*	Alias	Oligo FWD	Oligo REV	Sonde UPL
*Abcg5*	ABCG5	cggagagttggtgttctgtg	caccgatgtcaagtccatgt	82
*Abcg8*	ABCG8	cagatgctggctatcataggg	ctgatttcatcttgccacca	13
*Acat2*	ACAT-2	agaagtccttcagagagccaaa	cactggcttgtcgagtagga	50
*Acaca*	ACC	acagagatggtggctgatgtc	gatccccatggcaatctg	4
*Actb*	Actb (control)	cccgcgagtacaaccttct	cgtcatccatggcgaact	17
*Mlxipl*	ChREBP	aatcccagcccctacacc	ctgggaggagccaatgtg	10
*Gapdh*	Gapdh (control)	ccctcaagattgtcagcaatg	agttgtcatggatgaccttgg	73
*Hmgcr*	HMGCoAR	caaccttctacctcagcaagc	acagtgccacacacaattcg	80
*Hprt*	Hprt (control)	gaccggttctgtcatgtcg	acctggttcatcatcactaatcac	95
*Ldlr*	LDL-R	tgctactggccaaggacat	ctgggtggtcggtacagtg	16
*Lrp1*	LRP-1	aatcgagggcaagatgacac	ccagtctgtccagtacatccac	81
*Pcsk9*	PCSK9	ccagtgtccacgcttcct	gatgccatgctccttgattt	1
*Scarb1*	SR-B1	ggtgcccatcatttaccaac	gcgagccctttttactacca	71
*Srebf2*	SREBP2	gtgcagacagtcgctacacc	aatctgaggctgaaccagga	62

### Statistical analyses

All data are expressed as means ± standard error of the mean (SEM). Statistical comparisons were performed using a two-way analysis of variance (ANOVA) for non-repeated measures, using diet and time as main effects. Fisher’s post-hoc test was used in the event of a significant (*P* < 0.05) F ratio.

## Results

### Anthropometric parameters and food intake

Body weight was similar for all dietary groups at the beginning of the experimental period (Table 3). Body weight was not statistically modified by the obesogenic diets after 2 weeks of treatment. However, it was significantly higher (*P* < 0.01) at 6 weeks. Hence, body weight was greater (*P* < 0.01) in rats fed with the HFD and WD than those submitted to the SD. Average food intake in g/d was lower (*P* < 0.01) in rats fed the HFD and WD as compared to rats fed the SD. Food intake in g/d was also lower (*P* < 0.05) in rats fed the HFD as compared to rats fed the WD (Table 3). When food intake was calculated in kcal/d, animals treated with the HFD and WD displayed higher values (*P* < 0.001) than those fed with the SD. The sum of the 3 visceral fat pad weights as well as the subcutaneous fat deposit were significantly (*P* < 0.05) higher in rats fed the HFD and WD compared to rats fed the SD after 2 (*P* < 0.05) and 6 weeks (*P* < 0.001; Table 3). HFD and WD resulted in similar body fat accumulations.

**Table 3:** Anthropometric parameters and food intake

	2 weeks			6 weeks		
	SD	HFD	WD	SD	HFD	WD
Initial bodyweight (g)	200 ± 3	199 ± 4	198 ± 3	199 ± 3	194 ± 3	192 ± 3
Final bodyweight (g)	343 ± 4	348 ± 6	339 ± 6	509 ± 7 ^†††^	549 ± 17 ^†††**^	556 ± 13 ^†††**^
Food intake (g/day)	28.5 ± 0.6	20.9 ± 0.3^***^	24.3 ± 0.3^***□□□^	31.2 ± 0.6 ^†††^	21.9 ± 0.4 ^†††***^	26.2 ± 0.6 ^†††***□□□^
Food intake (kcal/day)	82 ± 2	109 ± 1^***^	113 ± 2^***□^	90 ± 1 ^†††^	114 ± 2^*** †††^	123 ± 3 ^†††***□^
Abdominal fat pad weight (g)	11.7 ± 0.7	17.6 ± 1.0^*^	18.2 ± 1.0^*^	33.2 ± 1.7 ^†††^	48.6 ± 2.5 ^†††***^	52.1 ± 3.0 ^†††***^
Subcutaneous fat pad weight (g)	1.7 ± 0.2	2.6 ± 0.2^**^	2.8 ± 0.4^***^	4.9 ± 0.3 ^†††^	7.2 ± 0.6 ^†††**^	7.9 ± 0.9 ^†††***^

Values are mean ± SEM with 8-10 rats per group: *SD* standard diet; *HFD* high fat diet; *WD* western diet. * Significantly (*P* < 0.05) different from SD; ** *P* < 0.01; *** *P* < 0.001; □ significantly (*P* < 0.05) different from HFD; □□□ *P* < 0.001; ††† significantly (*P* < 0.001) different from the two-week values, respectively

### Liver and plasma triglycerides and cholesterol

**Fig. 1:**
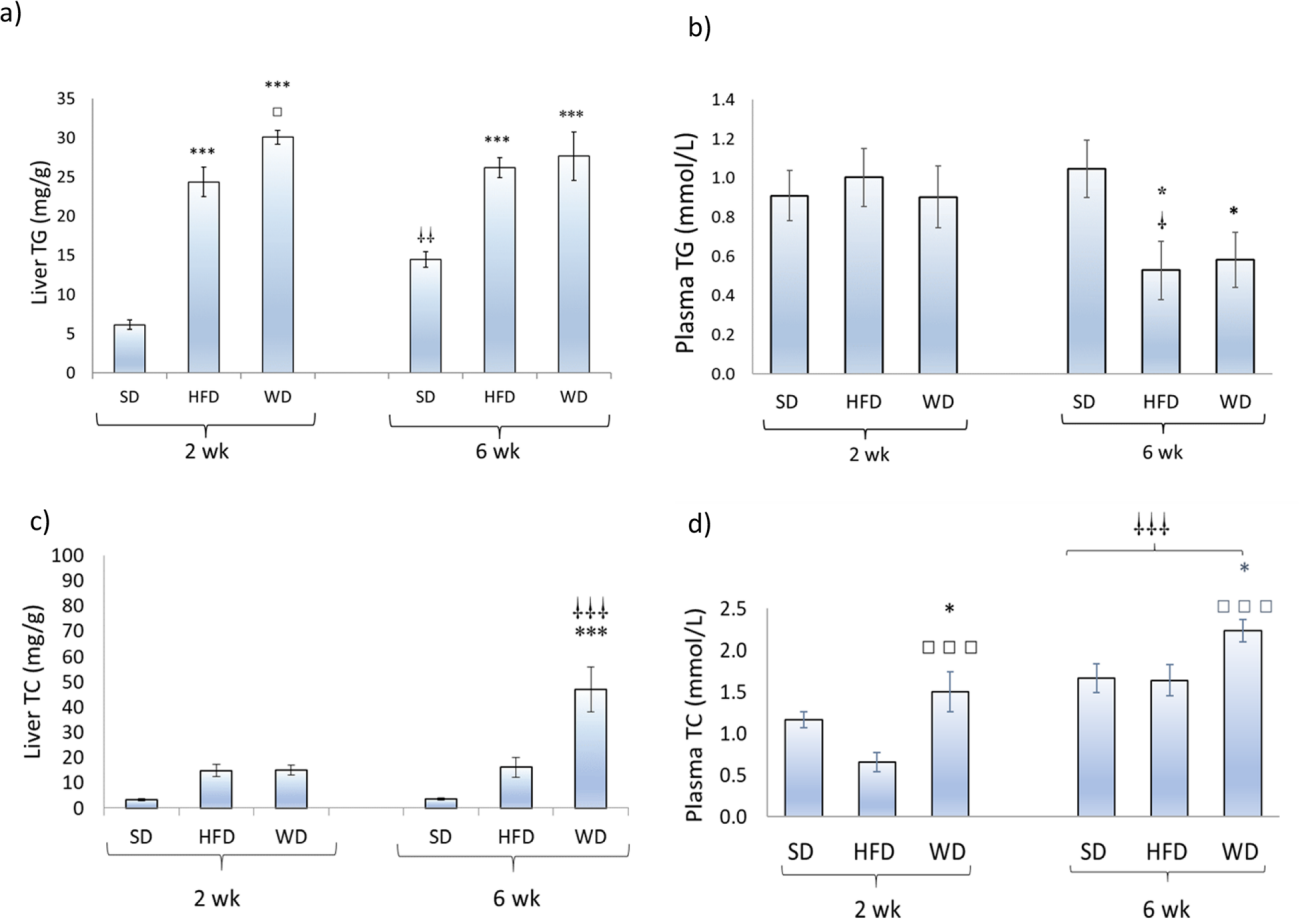
Liver and plasma triglyceride (TG) and total cholesterol (TC) concentrations in rats (*n* = 8–10) fed a standard (SD), high fat (HFD), or a western (WD) diet for two and six weeks. * Significantly (*P* < 0.05) different from SD; *** *P* < 0.001; □ significantly (*P* < 0.05) different from HFD; □□ *P* < 0.01; □□□ *P* < 0.001; □ significantly (*P* < 0.05) different from the two-week values, respectively; □□ *P* < 0.01

Liver TG accumulation was significantly higher (*P* < 0.001) in rats fed both the HFD and the WD after 2 as well as after 6 weeks (Fig. 1a). Liver TG levels were even slightly higher (*P* < 0.05) in WD than in HFD-fed animals after the 2-week feeding period. Liver TG levels were higher (*P* < 0.01) after 6 weeks than after 2 weeks in the SD-fed group. On the other hand, HFD and WD did not affect plasma TG levels after 2 weeks of feeding but resulted in a slight and significant (*P* < 0.05) decrease after 6 weeks of feeding (Fig. 1b).

Although some tendencies (*P* < 0.1) can be observed, liver cholesterol concentrations were not changed significantly (*P* > 0.05) by the diets with the exception of the group of animals fed the WD for 6 weeks that showed significantly (*P* < 0.01) higher levels compared to SD and HFD fed rats (Fig. 1c). On the other hand, plasma total cholesterol levels were higher (*P* < 0.05) in WD compared to SD and HFD fed animals after 2 and 6 weeks (Fig. 1d). No significant difference in plasma total cholesterol levels was found between rats fed the SD and the HFD. Overall, plasma total cholesterol levels were higher (*P* < 0.05) with the longer diet duration (Fig. 1d).

### Liver molecular markers

**Fig. 2:**
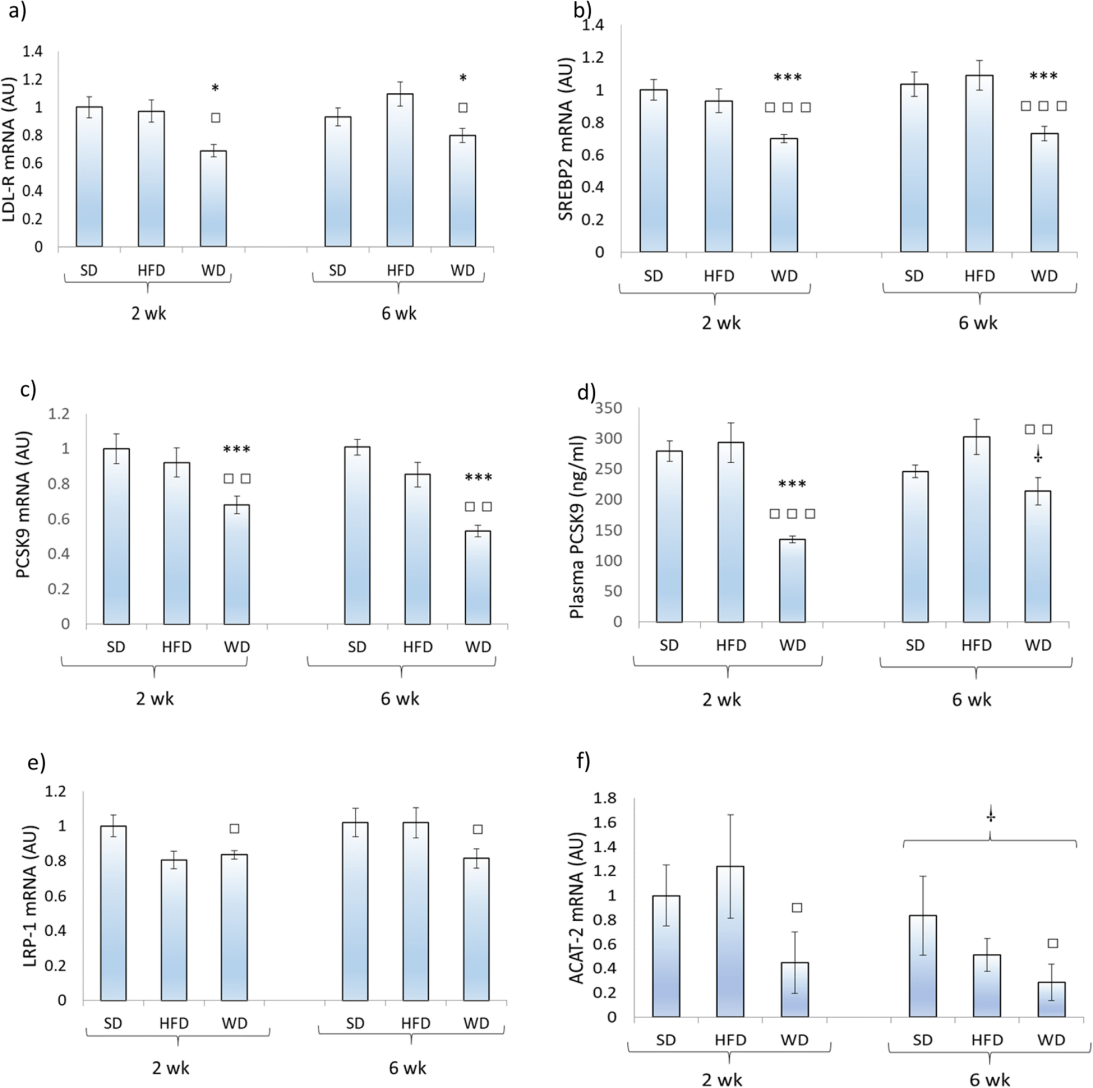
Hepatic gene expression of low-density lipoprotein receptor (LDL-R), proprotein convertase subtilisin/kexin type 9 (PCSK9), low-density lipoprotein-receptor-related protein 1 (LRP-1), sterol regulatory element binding protein 2 (SREBP2), acyl-CoA cholesterol acyltransferase-2 (ACAT-2), and plasma PCSK9 concentrations in rats (*n* = 9–10) fed a standard (SD), high fat (HFD), or a western (WD) diet for 2 and 6 weeks. * Significantly (*P* < 0.05) different from SD; *** *P* < 0.001; □ significantly (*P* < 0.05) different from HFD; □□ *P* < 0.01; □□□ *P* < 0.001; □ significantly (*P* < 0.05) different from the two-week values, respectively

As presented in Fig. 2a, rats submitted to the WD for 2 weeks depicted lower LDL-R gene expression levels as compared to SD (*P* < 0.05) and HFD (*P* < 0.05) animals. LDL-R mRNA levels were also lower (*P* < 0.05) in rats fed the WD compared to those fed the SD after 6 weeks. The same pattern of response was observed for the transcription factor SREBP2 (*P* < 0.001) that regulates both LDL-R and PCSK9 (Fig. 2b). Similarly, PCSK9 mRNA expression was lower (*P* < 0.01) in rats fed the WD as compared to animals fed the SD and HFD after 2 and 6 weeks (Fig. 2c). This pattern of response was similar for plasma PCSK9 concentrations after 2 weeks, while WD-fed animals depicted lower levels as compared to HFD-fed rats after 6 weeks (Fig. 2d). The transcription of LRP-1, a member of the LDL-R gene family, was also significantly (*P* < 0.05) lower after 2 and 6 weeks in animals submitted to the WD in comparison to animals fed the SD (Fig. 2e). Gene expression of ACAT-2 was also lower (*P* < 0.05) following 2 and 6 weeks of WD compared to SD, where the 6-week treatment showed even lower levels than the 2-week treatment (Fig. 2f).

**Fig. 3:**
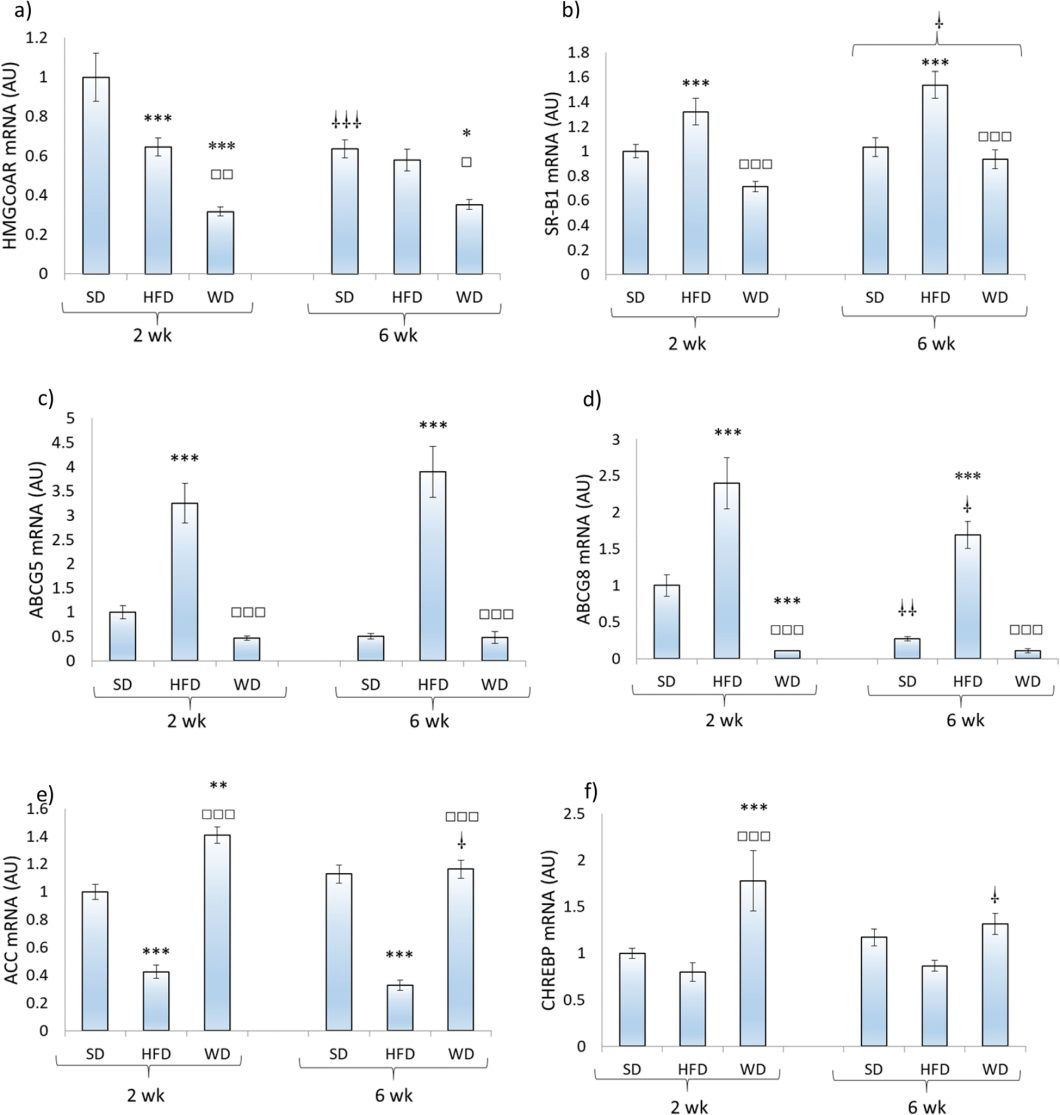
Hepatic gene expression of 3-hydroxy-3-methyl-glutaryl-CoA reductase (HMGCoAR), scavenger receptor class B member 1 (SR-B1), ATP-binding cassette protein G5 (ABCG5), ATP-binding cassette protein G8 (ABCG8), acetyl-CoA carboxylase (ACC), and carbohydrate-response element binding protein (ChREBP) in rats (*n* = 7–10) fed a standard (SD), high fat (HFD), or a western (WD) diet for 2 and 6 weeks. * Significantly (*P* < 0.05) different from SD; ** *P* < 0.01; *** *P* < 0.001; □ significantly (*P* < 0.05) different from HFD; □□□ *P* < 0.001; □ significantly (*P* < 0.05) different from the two-week values, respectively; □□ *P* < 0.01; □□□ *P* < 0.001

Transcripts of HMGCoAR, the main enzyme of cholesterol synthesis, were decreased (*P* < 0.001) after 2 weeks in rats fed the HFD and the WD while after 6 weeks lower levels (*P* < 0.05) were found only in rats fed the WD (Fig. 3a). Animals fed the SD depicted a lower level of gene expression for HMGCoAR after 6 weeks in comparison to the one observed after 2 weeks. Gene expression of SR-B1, the transporter responsible for the exchange of cholesterol from HDL particles to the liver, was significantly (*P* < 0.001) higher in HFD-fed rats compared to SD- and WD-fed animals in both feeding durations (Fig. 3b). A small but significant (*P* < 0.05) effect of overall diet duration was also found with higher values observed after 6 weeks. Gene expressions of ABCG5 and ABCG8 were significantly (*P* < 0.01) higher among rats fed the HFD in comparison to those submitted to 2 and 6 weeks of the other diets (Fig. 3c, d). Gene expression of ACC, an important enzyme regulating the first steps of de novo lipogenesis, was higher in rats fed the WD compared to rats fed the two other diets for 2 weeks (Fig. 3e). After 6 weeks of treatment, ACC transcription was also higher (*P* < 0.001) in rats fed the WD than in those treated with the HFD. ACC mRNA levels were also lower (*P* < 0.001) in HFD- than in SD-fed rats after 2 and 6 weeks. Gene transcription of ChREBP, the transcription factor involved in regulating mediators of de novo lipogenesis, was highly stimulated (*P* < 0.01) among rats fed the WD compared to those treated with the other dietary regimen for 2 weeks (Fig. 3f). This response was not observed after 6 weeks of treatment.

## Discussion

The main finding of the present study is that animals fed a WD (high fat + high sucrose) for only 2 weeks depicted lower gene expression of several key markers of liver cholesterol metabolism including LDL-R, PCSK9, SREBP2, and HMGCoAR compared to animals fed a SD or a HFD. The same response was also observed for PCSK9 measured in plasma. These findings were not observed when rats were fed the HFD. The 2-week WD also resulted in higher plasma total cholesterol levels. The HFD and even more so the WD promptly (2 weeks) induced excessive TG accumulation in the liver. These results indicate that the WD, which does not contain higher levels of cholesterol, disturbs the mechanisms involved in the regulation of cholesterol metabolism in the liver. The current results indicate that the enrichment in sucrose (50% glucose and 50% fructose) and fat of the WD promptly and potently promoted TG accumulation in the liver. Hence, this effect may be put forward as the element responsible for the alterations observed in the regulation of cholesterol metabolism. From a clinical perspective, these results suggest that high plasma cholesterol levels may be linked to lipid metabolism in the liver.

The first observation of the present study is that short-term (2 weeks) exposure to either a HFD (60% fat) or a WD (40% fat + 35% sucrose (17.5% fructose)) induced important increases in the liver TG levels and this effect remained stable after 6 weeks of treatment. An early accumulation of lipids in the liver in response to high-fat feeding has been previously observed [11, 15, 16]. This response has been interpreted as if the liver was acting as a buffer to protect the other organs from a potentially deleterious surge of lipids [11, 17]. Accordingly, plasma TG levels were not changed after 2 weeks of HFD or WD. An additional finding of the present study is that liver TG accumulation after 2 weeks was even higher in the WD than in the HFD. Fructose contained in the WD is a strong inducer of hepatic lipogenic enzymes [18]. Therefore, the present increases in gene expression of ACC and ChREBP measured in the liver of animals submitted to 2 weeks of WD support the assumption that de novo lipogenesis is associated with the excessive accumulation of fat in this organ.

In addition to resulting in a rapid increase in liver TG accumulation, the short 2-week period of feeding applied in the present study has the advantage of preceding significant increases in body weight gain and the systemic development of metabolic complications associated with longer exposure to obesogenic diets. In support of this, the body weight observed among animals fed the HFD and WD was similar to the body weight observed among rats fed the SD diet after 2 weeks. Body weight was approximately 50 g higher among the animals fed the obesogenic diets after 6 weeks. Moreover, the sum of abdominal and subcutaneous fat accumulation was approximately 3 times higher after 6 weeks of the obesogenic diets than it was after only 2 weeks (7 vs 20 g), all while liver TG levels were similar after 2 and 6 weeks. The present results support the validity of selecting such an experimental design where animals are submitted to 2 weeks of obesogenic diets. This is reinforced by the fact that despite no change in body weight was detected, hepatic lipid metabolism was strongly altered by the HFD and WD. For this reason, the present discussion is centered on results derived from the 2-week feeding condition.

Using this experimental design, the present study aimed to gather evidence for the existence of an association between a rapid increase in liver TG among rats submitted to short-term (2 weeks) obesogenic diets and disturbances in gene expression of key regulators of cholesterol metabolism in the liver. The main finding of the present study was that feeding animals with a WD for both 2 and 6 weeks resulted in a decrease in gene expression of three important and related markers of liver cholesterol metabolism: LDL-R, PCSK9, and the transcription factor SREBP2. The combined action of these 3 molecules regulates transport of highly atherogenic LDL particles. With approximately 70% of its expression carried out in the liver, it is clear that LDL-R is crucial for the clearance of LDL-cholesterol particles from circulation [19]. Therefore, the increase in plasma total cholesterol observed in rats fed a WD for 2 or 6 weeks could be the result of the decrease in gene expression LDL-R in the liver. While the present data strongly support the physiological relevance of this mechanism; the importance of other pathways involved in the regulation of circulating cholesterol levels will also require further attention in future studies.

The results also showed that the levels of mRNA and plasma PCSK9 (a protease that down-regulates LDL-R protein expression [20–22]) are reduced at 2 and 6 weeks after WD feeding. This could have normally contributed to promoting the clearance of plasma cholesterol by increasing LDL-R protein levels. However, because of the decrease in upstream gene expression of SREBP2, a transcription factor that regulates the expression of both LDL-R and PCSK9 [23, 24], the reduction in PCSK9 levels did not positively affect the expression of LDL-R. Consequently, plasma total cholesterol remained high in rats fed the WD at 2 and 6 weeks. Similarly, gene expression of LRP1, responsible for the removal of circulating lipoprotein remnants enriched in cholesterol [25], was reduced in rats fed the WD, thus potentially contributing to the increased plasma cholesterol levels. In addition, cholesterol transport from HDL to the liver is carried out through its interaction with receptor SR-B1 [26] whose gene expression remained unchanged by the WD while being modulated by the HFD. All of these responses may have contributed to the decrease in cholesterol uptake by the liver and, in turn, promoted its increased concentration in the circulation.

Along with LDL-R and PCSK9, the expression of HMGCoAR, the key marker of cholesterol synthesis in the liver, is regulated by the transcription factor SREBP2 [23, 24]. Accordingly, SREBP2 and HMGCoAR mRNA levels were highly reduced in rats fed the WD. At the same time, mRNA expression of ACAT-2, which is involved in cholesterol esterification was reduced in rats fed the WD compared to rats submitted to the HFD. Taken together, these results support the hypothesis that in the liver, a short-term WD promptly yields a large increase in the TG content and, in turn, impairs pathways involved in regulation of cholesterol metabolism.

If liver cholesterol uptake from the circulation is reduced under the present WD, the next question is what can trigger this response? The accumulation of cholesterol in the liver may be an important factor inducing the reduction in gene expression of key markers of the LDL-R pathway and, in turn, the increase in plasma cholesterol levels [23]. Liver cholesterol concentrations show a tendency (*P* < 0. 1) to be higher after 2 weeks of both the HFD and WD, a tendency that became highly significant for the WD after 6 weeks. In this regard, it is noticeable that gene expression of HMGCoAR was lowered in rats fed the HFD and even more so in rats fed the WD for 2 weeks. This response may have been stimulated by a transient rise of cholesterol levels in the liver. In addition, the low mRNA levels of ABCG5/G8 transporters (responsible for the exportation of cholesterol in the bile ducts) in rats fed the WD might have contributed to transient higher liver cholesterol levels in these animals. However, this association does not hold for the HFD-fed animals since ABCG5/G8 mRNA levels were increased in these groups of rats. It is likely that other mechanisms such as fecal excretion of biliary acids [27] or increased synthesis and secretion of VLDL are involved.

Other important questions that emerge from the present results are what factors alter the expression of key metabolic markers of hepatic cholesterol homeostasis in response to 2 weeks of WD, and why does the response in animals submitted to the HFD not reach the same extent? It must be taken into account that after 2 weeks, hepatic TG levels were higher in WD- than in HFD- fed rats, and this might have interfered with cholesterol metabolism. It is also possible that the different fat composition of the two diets might have played a role. A key element of the WD diet is its content of fructose. Fructose has been reported to increase protein levels of all de novo lipogenesis enzymes during its conversion into triglycerides [28, 29]. The WD-induced lipogenesis necessitates the synthesis of malonyl-CoA, which is not the case for dietary fat. Malonyl-CoA is a potent inhibitor of mitochondrial oxidation [30]. By doing so, increased malonyl-CoA levels may have indirectly contributed to the higher level of liver fat accumulation in animals submitted to the WD. A second interaction between fructose and cholesterol metabolism is that lipogenesis and cholesterol synthesis pathways both use acetyl CoA as a molecular precursor and both use the reducing equivalent NADPH as a sole source of energy. Although other factors may be involved, this may have contributed to the lower gene expression of HMGCoAR found in rats treated with the WD compared to rats fed the HFD for 2 weeks. In one of the rare studies evoking the potential link between fructose and hepatic cholesterol metabolism, Ichigo et al. [12] reported a decrease in ABCG5/G8 gene expression in rats submitted to 12 days of high fructose compared to rats fed a high glucose diet. Although comparisons between this study and the present one remain difficult, these authors did hypothesize that a fructose diet might affect genes involved in regulation of cholesterol metabolism.

Although the precise mechanism linking liver fat accumulation to the disruption of liver cholesterol metabolism is beyond the scope of the present study, the results clearly indicate that a short-term WD disturbs hepatic cholesterol metabolism while promoting a rapid increase in total cholesterol circulating levels. Results derived from previous reports show that cholesterol homeostasis in the liver may be perturbed by activities of other metabolic pathways. For instance, Kemper et al. [31] reported that the ingestion of an ester of *B*-hydroxybutyrate in rats promoted an increase in LDL-R and SREBP2 protein levels. More recently, Rana et al. [32] reported that the cell surface protease-activated receptor PAR2 is a factor linking gene expression of several markers of cholesterol metabolism (i.e. LDL-R, SR-B1) to increased TG levels in the liver. This effect could potentially be mediated through the action of the de novo lipogenesis pathway. On a long-term basis, fructose has been highly associated with the development of liver steatosis in humans [33]. Several pathways modulating hepatic cholesterol metabolism are dysregulated in response to NAFLD [7]. Hence, the difficulty in determining the direct association between liver fat accumulation and impairments in hepatic cholesterol homeostasis is increased by the knowledge that NAFLD is also associated with several metabolic disorders (i.e. inflammation and insulin resistance, etc.), which, in turn, alter cholesterol metabolism in the liver.

### Strengths and limitations

It is currently accepted that the fructose enrichment of WD promotes de novo lipogenesis in the liver. The present data validated the use of a 2-week WD as a model of isolated hepatic steatosis yielding a rapid increase in circulating cholesterol levels. Thus, it supports a new concept inferring that an excessive accumulation of lipids interferes with the regulation of cholesterol metabolism in the liver. Although protein levels were not measured in the present study, mRNA levels of several key markers of the LDL-R pathway display the same pattern of responses.

## Conclusions

From a clinical point of view, the present findings suggest that liver fat accumulation, by disrupting the proper regulation of cholesterol pathways in the liver, contributes to an increase in plasma cholesterol levels. Further investigations should clarify the mechanisms linking fat accumulation in the liver to cholesterol pathways.

## Data Availability

All data and biological material will be available if needed.
